# Comparison of dose distributions hippocampus in high grade gliomas irradiation with linac-based imrt and volumetric arc therapy: a dosimetric study

**DOI:** 10.1186/s40064-015-0894-x

**Published:** 2015-03-05

**Authors:** Emine Canyilmaz, Gonca Dilek Hanedan Uslu, Fatma Colak, Burcin Hazeral, Emel Haciislamoglu, Ahmet Yasar Zengin, Ahmet Sari, Adnan Yoney

**Affiliations:** Department of Radiation Oncology, Faculty of Medicine, Karadeniz Technical University, Trabzon, Turkey; Department of Radiation Oncology, Kanuni Research and Education Hospital, Trabzon, Turkey; Onkomer Oncology Center, İzmir, Turkey

**Keywords:** Glioma, Hippocampus, IMRT, Partial brain radiotherapy, VMAT

## Abstract

The aim of this study was to assess the feasibility of sparing contralateral hippocampus during partial brain radiotherapy in high grade gliomas. 20 previously treated patients were replanned to 60 Gy in 30 fractions with sparing intensity-modulated radiotherapy (IMRT) and volumetric modulated arctherapy (VMAT) using the following planning objectives: 100 % of PTV covered by 95% isodose without violating organs at risk (OAR) and hot spot dose constraints. For each, standard intensity-modulated radiotherapy (IMRT) plans were generated, as well as sparing IMRT and VMAT plans which spared contralateral (hemispheric cases) hippocampus. When the three plans were compared, there was equivalent PTV coverage, homogeneity, and conformality. Sparing IMRT significantly reduced maximum, mean, V20, V30 and V40 hippocampus doses compared with standart IMRT and VMAT (p < 0.05). VMAT significantly reduced maximum left lens and mean eye doses compared with standart IMRT and sparing IMRT (p < 0.05). Brainstem, chiasm, left and right optic nerves, right eyes and lens doses were similar. VMAT significantly reduced monitor units compared with standart IMRT and sparing IMRT (p < 0.05). It is possible to spare contralateral hippocampus during PBRT for high grade gliomas using IMRT. This approach may reduce late cognitive sequelae of cranial radiotherapy.

## Introduction

The prognosis of the patients with high grade gliomas had improved with the combination of radiotherapy and chemotherapy. While expected life time has improved, the ratio of late side effects of radiotherapy has also increased (Stupp et al. 2009). Side effects are more frequent in children after partial brain radiotherapy (PBRT). These side effects include cognitive dysfunctions, endocrine dysfunctions, visual loss, hearing loss, myelopathy, vasculopathies and the induction of secondary tumours including gliomas (high and low grade), sarcomas and meningiomas (Merchant et al. 2005; Nandagopal et al. 2008; Kondoh et al. 2003; Douw et al. 2009; Crossen et al. 1994; Kortmann et al. 2003) Clear dose–volume relationships exist for many of these late adverse events. However, no clear dose–response relationships have been proven for cognitive dysfunction and secondary tumour induction (Merchant et al. 2005; Marks et al. 1981). These side effects are seen less often in elderly patients (Douw et al. 2009). Nevertheless, without necrosis, radiation induced cognitive dysfunction and leuko-encephalopathy are the most often complications in patients with long-term survival (Crossen et al. 1994). This clinical presentation is different than radiation necrosis in terms of clinic-radiology and pathology. The most clinically dramatic side effect is radiation induced dementia, and it is characterized by memory loss, attention deficit and emotional changes (Armstrong et al. 1995; Vigliani et al. 1997). Frequent memory loss and other cognitive deficits are induced by radiotherapy in the hippocampus and limbic system. According to clinical and pre-clinical study results, the key role of neurocognitive deficit pathogenesis induced by cranial radiotherapy takes place in the neural stem cell compartment of the hippocampus (Raber et al. 2004; Nagai et al. 2000; Peissner et al. 1999; Roman and Sperduto 1995). The hippocampus is a paired brain structure, located in the ventromedial part of the temporal lobes, laying lateral to the temporal horn of the lateral ventricle. Bilateral and unilateral radiation injury of the hippocampus is known to alter learning and memory formation (Tofilon and Fike 2000). Clinical data defining a clear dose–response effect does not exist but there is a suggestion that more than 30 Gy to the temporal lobe may be relevant (Armstrong et al. 2010).

Intensity modulated radiotherapy (IMRT) can allow dosage increase in high grade gliomas as well as reduce late toxicity due to radiotherapy. According to a retrospective analysis that compared IMRT with conformal radiation therapy, IMRT provided equal overall survival without prognosis change, and it decreases acute and late neurotoxicity (Merchant et al. 2005). In dosimetric studies of high grade gliomas, when IMRT is compared with three-dimensional conformal irradiation, IMRT is superior in limiting exposure for organs at risk (OAR) and allows for the planned target volume coverage (Narayana et al. 2006; MacDonald et al. 2007). As an extension of IMRT, volumetric modulated arc therapy (VMAT) is currently in use for the treatment of many cancers. Gantry arc-based IMRT delivery methods involve the gantry rotating around the patient while the multileaf collimator (MLC) leaf positions, dose rate and gantry speed are varied simultaneously (Otto 2008).

This current dosimetric study was conducted to evaluate the preservability of the contralateral hippocampus, in case of ipsilateral hippocampus invasion by a tumor during PBRT in high grade hemispheric gliomas.

## Methods and materials

We identified 20 patients with high-grade glioma who previously have been treated in our department with PBRT. Patient and tumor characteristics are shown in Table 1. In our study, we used CT scans of 20 patients diagnosed with hemispheric high grade glioma in our clinic after approval of ethical committee. Patients were immobilized in the supine position using an individualized thermoplastic mask. CT and magnetic resonance imaging scans were performed, both with a 2.5-mm slice thickness, and were used to aid tumor delineation. We reloaded CT scans that were used in the treatment of the patients and archived in CT-simulator into the Eclipse treatment planning system. Target volume and OAR identified in the CT scans of all patients were contoured by a radiation oncologist to prevent personal contouring differences that could skew the study results. Two researchers, one of which is a radiologist, evaluated the contouring. We made IMRT planning suitable to dosage constraints in our study using Eclipse TPS in our department. Afterwards, CT scans were transferred to another treatment center to make VMAT planning.

**Table 1:** **Patient and tumor characteristic**

**Total number of patients**	**20**
Age	
Median	53 years
Range	31–75 years
Sex	
Male	13
Female	7
CTV size	
<6 cm	6
>6 cm	14
Location	
Frontal	2
Parietal	5
Temporal	5
Temporo-parietal	3
Fronto-temporal	2
Temporo-oksipital	3
Contralateral hippocampal volume cm^3^	
Median	2.6
Range	1.9-3.4

For each patient, two intensity-modulated radiotherapy treatment plans and VMAT plans were generated using the eclipse Version 10.1 treatment planning system. All IMRT and VMAT plans were generated using 6-mv for a linear accelerator and 120-leaf multileaf collimator (MLC) (5-mm width leaves over target extent). For both techniques, final dose calculations were performed using the single pencil beam algorithm in Eclipse. The DVH calculations were also performed in Eclipse.

The standart IMRT plan, designated standard, did not include hippocampus as avoidance structures in optimisation criteria. The sparing IMRT and VMAT plans, designated sparing, did introduce hippocampus as avoidance structures. Optic nerve, chiasma, brain stem, eyes and lenses were the structures contoured in the study as OAR. The hippocampus was contoured on T1-weighted MRI axial sequences. The contralateral hippocampus was contoured by the reference of Gondi et al (Gondi et al. 2010). The baseline optimisation criteria for the high grade glioma cases are shown in Table 2.

**Table 2:** **Baseline optimization criteria for all plans**

**Structure**	**Optimisation criteria**
Initial phase (46 Gy in 23 fractions to PTV 46 Gy)	
PTV 46 Gy	95% to receive 46 Gy
Right eye	0% to receive 30 Gy
Left eye	0% to receive 30 Gy
Right lens	0% to receive 6 Gy
Left lens	0% to receive 6 Gy
Right optic nerve	0% to receive 42 Gy
Left optic nerve	0% to receive 42 Gy
Optic chiasm	0% to receive 42 Gy
Brainstem	0% to receive 42 Gy
Hippocampus	0% to receive 15 Gy
Boost phase (14 Gy in seven fractions to PTV 60 Gy)	
PTV 60 Gy	95% to receive 14 Gy
Right eye	0% to receive 5 Gy
Left eye	0% to receive 5 Gy
Right lens	0% to receive 4 Gy
Left lens	0% to receive 4 Gy
Right optic nerve	0% to receive 12 Gy
Left optic nerve	0% to receive 12 Gy
Optic chiasm	0% to receive 12 Gy
Brainstem	0% to receive 12 Gy
Hippocampus	0% to receive 5 Gy

In the traditional IMRT and VMAT plans, the prescription dose for phase I was 46Gy in 23 fractions delivered to PTV 46Gy, followed by a sequential cone down boost of 14Gy in seven fractions delivered to PTV 60Gy. For all plans, the following structures were contoured: gross tumor volume (GTV) (gross enhancing tumour and resection cavity as identified on postoperative MRI T1 post-contrast sequences), peri- tumouraloedema (as identified on postoperative MRI T2 fluid attenuated inversion recovery sequences), clinical treatment volume (CTV) 46Gy (GTV + peri-tumouraloedema + 2-cm anatomically constrained margins), PTV46Gy (CTV 46Gy + 5-mm margin), CTV 60Gy in IMRT plans (GTV + anatomically constrained 2-cm margin), PTV 60Gy in IMRT plans (CTV60Gy + 5-mm margin) (Chang et al. 2007; Nelson et al. 1993). Two different IMRT plans were performed for standart and sparing IMRT.The first IMRT plan set used seven beams (0°,51°,102°,153°,204°,255°,306°) isocentrically centered on the PTV for PTV 46. The second IMRT plan set used five beams isocentrically centered on the PTV for PTV 60. Five field IMRT plan special to each patient with angles according to the tumor location. All beams were collimatedt o minimize exposure to the hippocampus. The dose homogeneity goal was < 110° within the PTV. Achieve at least 100% coverage of PTV with the 95% isodose of 46 and 60 Gy without violating OAR maximum dose constraints.

The VMAT plans were generated using an in-house inverse planning approach, in which MLC-shaped fields were progressively added throughout a single 360 arc during optimization. The dose rate was between 0 and 600MU/min and gantry rotation between 0.0°/s and a maximum of 4.8°/s. The collimator was rotated to about 45° to 315° to minimize the contribution of the tongue-and Groove effect during treatment. The MLC constraints are included in the optimization, ensuring that the plan is always deliverable. Inhomogeneity corrections were not implemented for this in-house planning system and were therefore not used during optimization or final dose calculation. In VMAT planning, we used 2 arc plan to decide the most suitable treatment plan for PTV46 and PTV60. After failing to obtain dose homogeneity for PTV, we made 1 arc and 3 arc VMAT plans. As a result, we decided to use 2 arc VMAT planning because of highest PTV homogeneity. Both for PTV 46 and PTV 60; we used 45° collimation between 182°-178°, 178°-182° angles for the planning of patients with right hemispheric localization. We used 45°collimation between 178°-182° and 182°-178, for planning of patients with left hemispheric localization.

### Evaluation of treatment plans

A comparative analysis was then performed using the twenty computed tomography data sets. Dosimetric parameters were evaluated for target volumes and OARs. For PTV, the comparison parameters included maximum dose (D max), mean dose (D mean), D95%, conformality index (CI), and homogeneity index (HI). We evaluated D mean and D max for the hippocampus, Dmax dose for lens, eyes, optic nerves, brain stem, and chiasm for OARs.

The CI of the PTV was defined as:

$$ \mathrm{C}\mathrm{I}={\mathrm{V}}_{\mathrm{PTV}}\mathrm{x}\ {\mathrm{V}}_{\mathrm{TV}}/\mathrm{T}{{\mathrm{V}}_{\mathrm{PV}}}^2 $$

^V^_ PTV_^:Treatment volume for identified isodose, V^_TV_^:Volume of PTV, TV ^_PV_^2:Volume of PTV inside of treatment volume for identified isodose.^

The HI of the PTV was defined by using this formula:

$$ \mathrm{H}\mathrm{I}=\mathrm{D}5\%/\mathrm{D}95\% $$

D5%; Dose received by 5% of PTV, D95% Dose received by 95% of PTV (Ding et al. 2006; Lee et al. 2012).

### Statistical analysis

Dose-volume histograms (DVH) were used in the comparison of the target volume and at-risk organ doses in all the treatment plans. In the comparison of the data, if parametric conditions were provided, ANOVA post hoc was used, otherwise, the Kruskal-Wallis Test was used. In the paired group comparisons of quantifiable data, if parametric conditions were provided the Bonferroni Modified test was applied, otherwise the Mann-Whitney *U*-test was used. All statistical tests were two-sided, with a threshold for statistical significance of P < 0.05. Statistical analysis was carried out utilizing SPSS version 13.

## Results

Coverage of the treatment target (PTV60 Gy and PTV46 Gy in the high-grade glioma plans) were essentially identical in the Standard IMRT, Sparing IMRT, and VMAT plans, with the whole target receiving at least 95% of the prescription dose (D100 95%). All IMRT and VMAT plans were able to meet the constraints placed on the OAR, as well as PTV. All plans were optimised to keep the maximum dose within the target to <110% of the prescription dose (Dmax < 110%), and all plans were able to meet this objective. The PTV coverage, conformality, and homogeneity were equivalent with VMAT and IMRT. Comparing VMAT with all IMRT, maximum doses to the brainstem, chiasm, left and right optic nerves, right eyes and lens doses were similar. VMAT significantly reduced maximum left eye and lens doses compared with standart IMRT and sparing IMRT (p < 0.05). There was a statistically significant difference noted between the three plans in terms of monitor units (p < 0.05). The mean MU to treat a 2-Gy fraction was 1472 (1041-1834) with standart IMRT, compared with 1320 (1116-1993) sparing IMRT and with 994 (886-1150) VMAT. Lowest mean monitor unit value was received in VMAT planning despite two arc. These dosimetric outcomes are shown in Table 3.

**Table 3:** **Dosimetric comparison of Standard IMRT plan, Sparing IMRT plan and VMAT planfor PTV 46 and 60 plans**

	**Standard IMRT plan**	**Sparing IMRT plan**	**VMAT plan**	**p value**
PTV46 max dose (Gy)	46.9 (46.2-47.4)	47 (46.5-47.6)	47.5 (48.5-50.1)	0.14
PTV46 mean dose (Gy)	48.9 (48-50.4)	49.3 (48.3-50.8)	48.5 (47.9-50.4)	0.21
D 95 46 (Gy) mean SD	46 (45.7-46.3)	46 (45.8-46.3)	46.4 (45.8-46.8)	0.55
PTV60 max dose (Gy)	63.2 (60.7-62.2)	63.4 (60.7-62.1)	63.6 (61.2-62.6)	0.44
PTV60 mean dose (Gy)	61 (61-64.3)	61.2 (62.4-64.9)	61.4 (61.3-64.6)	0.23
D 95 60 (Gy)	60 (59.7-60.2)	60 (59.9-60.3)	60 (59.9-61.5)	0.65
HI (60 Gy)	1.03 (1.02-1.05)	1.03 (1.02-1.04)	1.03 (1.02-1.04)	0.34
CI (60 Gy)	1.2 (1-1.4)	1.2 (1.1-1.6)	1.2 (0.9-1.6)	0.48
MU (0-60 Gy)	1472 (1041-1834)	1320 (1116-1993)	994 (886-1150)	*0.01*
Right lens max	4.8 (2.5-9.3)	5.2 (2.8-9.3)	7.5 (4.1-10.8)	0.1
Left lens max	7.7 (4.6-10.3)	6.1 (3.8-9.8)	4.0 (3.7-9.9)	*0.001*
Right eye mean	8.3 (4-25.8)	9.4 (5.1-16.7)	10.3 (3.6-17.6)	0.45
Left eye mean	10.8 (4.2-19.7	9.4 (6.2-16.2)	8.4 (5.9-16.3)	*0.023*
Right optic nerve max	35.1(29.2-41.0)	35.7 (30.6-40.8)	32.2 (25.2-39.1	0.65
Left optic nerve max	36.1 (30.2-42.1)	37.9 (32.4-43.5)	33.2 (25.6-40.8)	0.55
Optic chiasm max	39.8 (33.7-45.6)	43.2 (37.9-48.5)	37 (29.6-45.2)	0.7
Brainstem max	51.1 (48.2-54.3)	50.9 (48.3-54.5)	50.1 (48.9-54.1)	0.44

**Figure 1:**
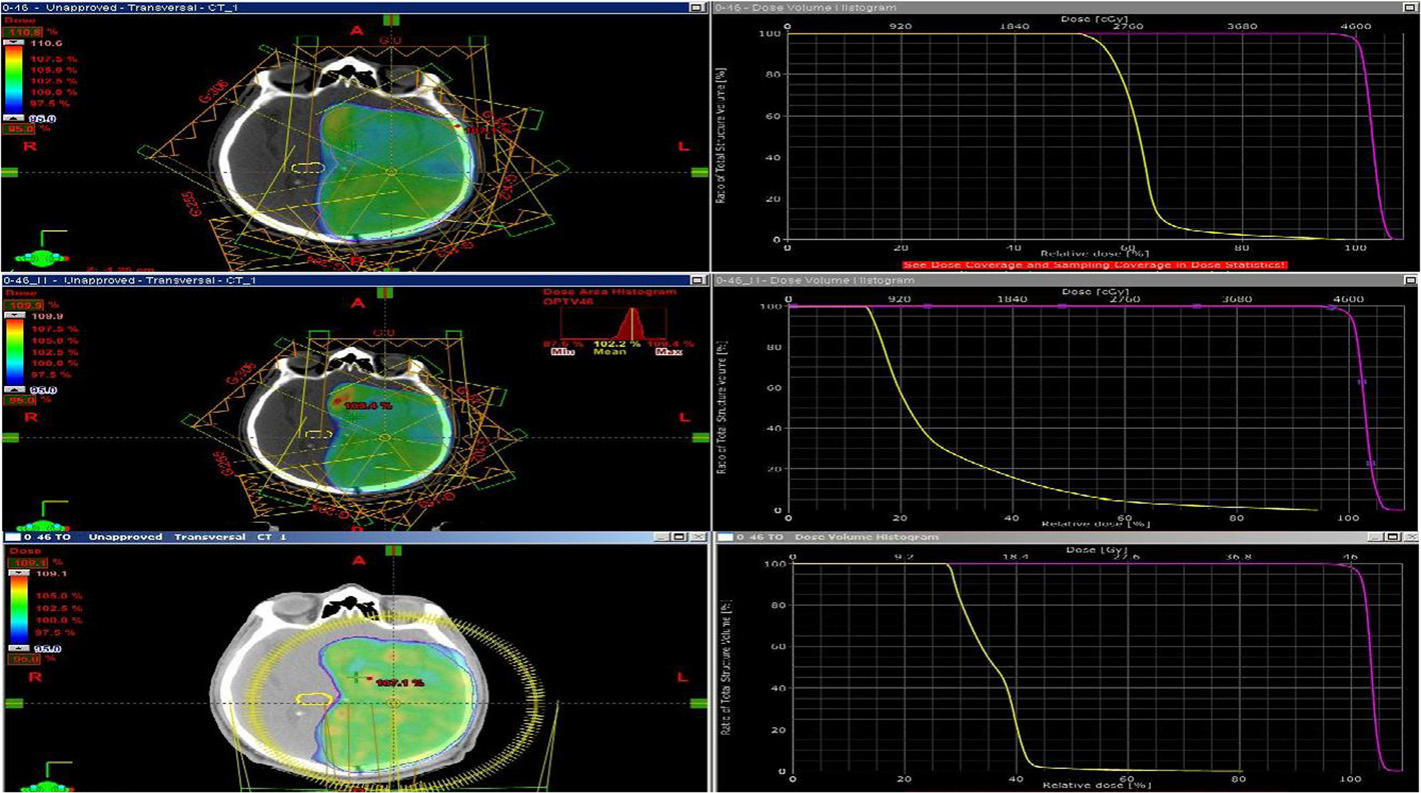
**Dose distrubutions and dose volume histograms.**

The lowest mean and max doses for the hippocampus were found in the sparing IMRT planning, and this result was statistically significant (p < 0.05). In V10, V20, V30, and V40 dose values for the hippocampus in standard IMRT planning, we observed a larger decrease in sparing planning comparing to VMAT planning (Table 4), (Figure 1).

**Table 4:** **Comparison of hippocampus between mean,max,V10, V20, V30, and V40 doses**

	**Standard IMRT Plan**	**Sparing IMRT Plan**	**VMAT plan**	**p value**
Hippocampus Mean dose (Gy)	32.4	15.07	17.2	0.001
Hippocampus Max Dose (Gy)	44.8	39.9	27.7	0.001
Hippocampus V10 mean dose (Gy)	100	80.4	92.4	0.002
Hippocampus V20 mean Dose (Gy)	98.9	17.3	38.7	0.001
Hippocampus V30 mean dose (Gy)	71.7	4.1	0	0.001
Hippocampus V40 mean (Dose Gy)	5	0.7	0	0.002

## Discussion

Cognitive and endocrine dysfunctions are both important and recognized problems after PBRT and complete brain radiotherapy in pediatric as well as mature patients (Merchant et al. 2005; Nandagopal et al. 2008; Kondoh et al. 2003; Douw et al. 2009). Standard treatment includes maximal safe surgical debulking and adjuvant radiotherapy, with chemotherapy for high grade glioma. Better results of glioma therapies with treatments in recent years make these side effects more visible (Stupp et al. 2009). For this reason, we should focus efforts to protect the cognitive function in this cohort of patients.

This study aims to show preservation of the hippocampus in high grade glioma patients during different planning techniques. In our study, we did not aim to preserve the hippocampus in the same hemisphere with the tumor, because in many cases the ipsilateral hippocampus is affected by the tumor directly or by tumor edema indirectly. Relapse risk is increased if the ipsilateral hippocampus is protected in high grade gliomas (Chan et al. 2002). As is known, relapses occur most often within two cm diameter of tumor origin in high grade gliomas. This is one of the main reasons for treatment failure as is shown in many reports (Marsh et al. 2011). In addition, the hippocampus, the limbic system, and neural stem cell compartments are structures that are present bilaterally in human body. Memory loss or other cognitive dysfunctions are not monitored in patients who have undergone temporal lobectomy due to persistent epilepsy (Di Gennaro et al. 2006; Giovagnoli et al. 2007). This gives rise to thought of one normal medial temporal lobe is enough for normal memory function. Therefore in this dosimetric study, we aimed to protect the contralateral hippocampus during PBRT.

The clinical tolerance of the hippocampus to radiation is not well described but likely to vary according to patient age, total dose, dose per fraction, time between fractions, and the distribution of dose within it. Functional representation in the hippocampus of the dominant and non-dominant hemispheres may not be interchangeable and therefore the optimal dose constraint may change and not simply depend on the laterality of the tumour. Johannesen et al. (Johannesen et al. 2003) researched changes in white matter using MRI scans of patients who received median 54 Gy cranial radiotherapy. According to the results of the mentioned study, there were changes noted in the white matter in T2 and FLAIR sequences of MRI above 29.2 Gy doses, however there was no change between 12.5-27.5 Gy. Also, researchers noted a relationship between grade 3 white matter changes in MRI T2 and FLAIR sequences with a lower quality of life as well as increase in side effects. In a pediatric study, Armstrong et al. (Armstrong et al. 2010) identified a relationship between memory loss and exposure of temporal lobe over 30 Gy. In a study conducted in mature adults, it is reported that 40 Gy or more exposure of brain leads to a change in the metabolic activity of the brain and effects neuro-cognitive functions (Hahn et al. 2009).

In a dosimetric study that comparing treatment plans in high grade gliomas while preventing contralateral hippocampus exposure, mean value for hippocampus in standard IMRT planning was 36.6 and it was found to be 15.8 Gy in sparing IMRT planning (Marsh et al. 2011). Another dosimetric study in the same group of patients reported a hippocampus mean value of 35.1 Gy, max dose 46.6 Gy in standard IMRT planning, and a mean dose 19.7 Gy, max dose 33.1Gy in sparing IMRT planning (Marsh et al. 2013). In another study consisting of eighteen patients with grade II and III glioma, the contralateral hippocampus mean dose was reported as 24.9 Gy. Despite no clinical data identifying optimal hippocampus mean dose, a dose between 15.8 and 24.9 Gy is generally considered for protecting neuro-cognitive functions (Pinkham et al. 2013). In our study, only sparing IMRT plan reached the determined mean and max dose values for the contralateral hippocampus. In standart IMRT plan contralateral hippocampus mean dose was 32.4 Gy and max dose was 44.8 Gy, while in VMAT plan mean dose was 17.2 Gy and max dose was 27.7 and in sparing IMRT plan mean dose was 15 Gy and max dose was 39.9 Gy. When the literature is examined, hippocampus mean dose values found in our study were lower compared to other studies.

In a dosimetric study comparing IMRT and VMAT planning in high grade gliomas, at OARs doses in IMRT planning were similar to our study however were higher for VMAT planning. In the same study, a reduction ratio of doses received by risky organs in VMAT planning was found statistically significant comparing to IMRT planning (Shaffer et al. 2010). In our study we were unable to show a statistically significant difference in doses received by at OARs in all three plans. However, contralateral hippocampus V10, V20, V30 and V40 dose values of the contralateral hippocampus of standard IMRT planning provided more reduction in sparing IMRT planning comparing to VMAT planning. We chose two arc plans instead of one arc in our study. In the two arc VMAT treatment plan, at OARs including the hippocampus were exposed to more dosage in comparison to other studies.

There was no statistically significant difference determined in regards to HI, CI, and PTV coverage. This data is similar to reports by Shaffer et al. (Shaffer et al. 2010). In the present study, monitor unit values of both IMRT planning and VMAT planning were found higher. In that study, seven fields for single PTV 60 in IMRT planning and single arc for single PTV 60 in VMAT planning were conducted (Shaffer et al. 2010). However in our study seven fields with standard angles for PTV 46 in IMRT planning and five fields identified according to tumor localization for PTV 60 were conducted. This explains higher mean monitor unit values in our study.

## Conclusion

This study aimed to show preservability of the hippocampus in different treatment techniques for patients who receive partial brain radiotherapy for high grade gliomas. While deciding for treatment plan of patients, the dose received by at OAR should be considered as treatment plan homogeneity. The lowest dose value for the hippocampus was identified in sparing IMRT planning for this study. This treatment plan can lower neuro-cognitive dysfunction, a late side effect of cranial radiotherapy. However, this should be supported with further studies that show the relationship between dose received by hippocampus during PBRT in high grade gliomas and neuro-cognitive dysfunctions.
